# Fixed-bearing versus high-flexion RP total knee arthroplasty (TKA): midterm results of a randomized controlled trial

**DOI:** 10.1186/s10195-018-0493-z

**Published:** 2018-07-17

**Authors:** Amit Chaudhry, V. K. Goyal

**Affiliations:** 1 Department of Orthopaedic Surgery, Military Hospital Kirkee, Pune, Maharashtra 20 India; 2 Department of Orthopaedic Surgery, Deen Dayal Upadhyay Hospital, New Delhi, India

**Keywords:** Total knee arthroplasty, Sigma RP-F mobile model, Midterm results

## Abstract

**Background:**

We compared the midterm results after total knee arthroplasty (TKA) using PFC Sigma RP-F mobile model with PFC Sigma PS fixed model.

**Materials and methods:**

In this randomized controlled trial, we analyzed 50 knees that underwent TKA with PFC Sigma RP-F and 60 knees with PFC Sigma PS fixed model. The follow-up period ranged from 76 to 104 months.

**Results:**

The knee score, function score, and radiographic evaluation were significantly not different between the two groups at final follow-up. No revisions, subluxations, dislocations, or infections were seen. Also, no radiographic evidence of component loosening, osteolysis, or malalignment was observed in any knee. The results for both groups show good patient satisfaction.

**Conclusions:**

The midterm clinical and radiographic results of the two prostheses did not show significant differences between the two groups.

**Level of evidence:**

Level of evidence is level II.

## Introduction

Total knee arthroplasty (TKA) has become a standard operative procedure to alleviate pain and restore function in patients with end-stage knee arthritis [1]. Both fixed and mobile bearing designs in TKA solve the main purpose of relieving pain and maintaining functional stability of the knee joint [2, 3]. Both designs show excellent survival rates and long-term durability [4–7]. The high success rate of this surgical procedure has led to the expectation of superior range of motion (ROM). High-flex (HF) activities such as sitting cross-legged on the floor, kneeling, and squatting are an integral part of many daily activities for the Asian population [1]. However, studies following conventional TKA reported maximal flexion not exceeding 110–120° in most cases [8].

Several factors influence postoperative ROM, including the diagnosis, preoperative ROM and deformity, age, gender, surgical technique, postoperative pain control and rehabilitation, and lifestyle [9]. Implant design is a major decisive factor affecting ROM after TKA. HF knee prostheses were introduced to provide superior improved ROM with higher flexion angle. However, results with HF implants have garnered mixed reviews. In some studies, HF-TKA showed superior ability for squatting, kneeling, and crossed-legged sitting, the three most important weight-bearing HF activities in the Asian population, requiring knee ROM between 111° and 165°, compared with conventional TKA [1]. Meanwhile, some recent studies also performed on Asian population reported an alarming, increased incidence of aseptic loosening of femoral components in HF-TKA and attributed it to HF activities done by those patients after HF-TKA. Additionally, the extra cost of these implants also needs to be weighed relative to their performance.

Therefore, we compared the posterior stabilized (PS) press-fit condylar (PFC) Sigma fixed (DePuy Orthopaedics Inc.) with the posterior stabilized PFC Sigma rotating platform (RP) mobile model (PFC Sigma RP-F mobile; DePuy Orthopaedics Inc., Warsaw, IN, USA) which has a thicker posterior femoral condyle to minimize polyethylene contact stresses during high flexion. This model also incorporates advantages of the mobile-bearing and high-flexion models [10].

Specifically, we aimed to compare patients managed with TKA with PS-fixed model and PFC Sigma mobile model in terms of (i) greater range of maximum flexion as outcome, (ii) functional outcome in terms of Knee Society pain and function scores, (iii) better durability of component fixation as reflected by radiographic outcome and rate of reoperation, and (iv) cost of implants relative to outcome, which is a more important consideration in developing countries such as ours.

## Materials and methods

This prospective, randomized controlled, single-blinded study was approved by the institution’s ethics committee. A total of 110 primary TKAs were performed for osteo- or rheumatoid arthritis on 84 patients between August 2007 and February 2010. PFC Sigma RP-F mobile (DePuy Orthopaedics Inc., Warsaw, IN, USA) or PS PFC Sigma fixed (DePuy Orthopaedics Inc.) model was used for the surgery in the cases. All patients were given full explanation of the study and potential advantages of one prosthesis design over the other. The exclusion criteria were ≥30° flexion contracture, ≥20° varus deformity, ≥10° valgus deformity, and ≥30 kg/m^2^ body mass index (BMI). Patients were divided into two groups according to the implant used, and for both groups patients were selected randomly through random number tables. All subjects recruited for the study had given written informed consent and were available for a minimum 6 years of follow-up.

All patients were above 45 years in age with clinically and radiologically established advanced stage of osteoarthritis/rheumatoid arthritis in which severe pain and functional disability was not relieved by other forms of treatment such as conservative therapy, arthroscopic lavages and debridement, etc.

In the PFC Sigma RP-F mobile model TKA group, there were 34 cases (10 male, 24 female) with mean age of 58.7 years (range 46–74 years). In the PS fixed model TKA group, there were 50 cases (14 male, 36 female) with mean age of 57.6 years (range 46–72 years). The follow-up period ranged from 76 to 104 months.

All operations were performed by the same surgeon using a standard medial parapatellar approach. Regional anesthesia was used in all patients. Extension and flexion gap balancing was performed, using the gap technique. Cement was used for fixation of the tibial and femoral components in all cases. Cementing was initiated on the tibia, followed by femur and patella. The cement was applied on the surface of the implants, resected bone surface, and posterior cut of the femur, while exerting pressure with fingers prior to fixation. When the cement was completely hardened with pressure applied to the axis of the joint longitudinally, the tourniquet was released. After hemostasis was obtained, the trial polyethylene component and extra cement were removed, and a real polyethylene component was inserted. The postoperative rehabilitation program was identical in both groups. Quadriceps femoris strengthening exercises were initiated from the 2nd postoperative day. Continuous passive motion (CPM) using a machine was allowed from the 3rd postoperative day, if straight leg raising was possible and quadriceps muscle strength was recovered [10].

Patients were clinically and radiologically evaluated using the Knee Society clinical rating system [11] and Knee Society radiographic evaluation and scoring system [12] preoperatively and at 1.5, 3, and 6 months and 1 year postoperatively, and yearly thereafter.

Statistical analysis was carried out using SPSS version 17 (SPSS Inc., Chicago, IL, USA). Power analysis was conducted taking into account maximum flexion angle difference 10°, standard deviation 20, significance level 0.05, *α* error = 0.05, *β* error = 0.2. Using appropriate formula, we found the minimum cases (surgeries to be performed) to be 63 in each group. We were able to perform slightly fewer surgeries in both groups (60 in PFC Sigma PS group, 50 in PFC Sigma RP-F group). A possible explanation for this attrition could be certain social factors such as financial constraints and belief in the outcome of the study due to randomization. Paired *t* test was used to assess differences between preoperative and postoperative values of all continuous outcome variables, including the variables for Knee Society score (KSS), ROM, flexion contracture, and maximum flexion angle. Differences of at least *p* < 0.05 (two-sided) were considered statistically significant (Table 1).

**Table 1:** General demographic features for all patients in the study

Model	PFC Sigma PS group (*N* = 50)	PFC Sigma RP-F group (*N* = 34)	*p*-Value
Knees operated	60	50	–
Female/male	36/14	24/10	–
Age, mean (years)	57.6	58.7	0.13
Range (years)	(46–72)	(45–74)	
Body mass index (BMI) (kg/m^2^)	25.1 (20.8–29.5)	25.7 (21.4–30)	0.84
Diagnosis			
Osteoarthritis	46	40	0.91
Rheumatoid arthritis	14	10	0.75
Patella resurfacing	20	30	0.11

## Results

Patients who satisfied the inclusion and exclusion criteria were recruited for the study. There were 24 men and 60 women in the study. Clinical KSS, functional KSS, and radiographic evaluation were performed in all patients.

The mean preoperative clinical and functional KSS for the PFC Sigma PS group were 28.5 points (range 6–51 points) and 14.3 points (range 0–35 points), respectively, and the average scores at final follow-up evaluation were 90.7 points (range 80–99 points) and 76.7 points (range 55–90 points), respectively. For the RP-F group, the average preoperative clinical and functional KSS were 26.6 points (range 5–49 points) and 15.4 points (range 0–35 points), respectively, and the average scores at final follow-up evaluation were 92.2 points (range 82–99 points) and 77.6 points (range 55–90 points), respectively.

The Knee Society knee score (KSKS) and Knee Society function score (KSFS) improved significantly between the preoperative and last follow-up evaluation in both groups: from 28.5 points to 90.7 points (*p* < 0.0001) and from 14.3 points to 76.66 points (*p* < 0.0001), respectively, in the PFC Sigma PS group and from 26.6 points to 92.2 points (*p* < 0.0001) and from 15.4 points to 77.6 points (*p* < 0.0001), respectively, in the RP-F group.

The mean fixed flexion deformity (flexion contracture) and mean maximum flexion angle preoperatively were 8.83° (range 0–20°) and 85.66° (range 40–115°), respectively, whereas the mean flexion contracture and mean maximum flexion angle at last follow-up were 2.0° (range 0–10°) and 114.62° (range 90–130°), respectively, in the PFC Sigma PS group. For the RP-F group, the mean flexion contracture and mean maximum flexion angle preoperatively were 8.20° (range 0–20°) and 91.20° (range 70–115°), respectively, whereas the mean flexion contracture and mean maximum flexion angle at last follow-up were 1.60° (range 0–10°) and 114.4° (range 110–130°), respectively.

Significant improvement in flexion contracture was observed in both groups (*p* < 0.0001 for both PFC Sigma PS and RP-F group). There was no significant difference in preoperative (*p* = 0.18) or postoperative (*p* = 0.91) maximum flexion angle between the groups.

Preoperative ROM was comparable in the two groups. Mean ROM significantly improved between the preoperative and last follow-up evaluations in both groups (*p* < 0.0001 for both PFC Sigma PS and RP-F groups). However, there was no significant difference in ROM postoperatively between the groups (*p* = 0.33).

Our results found no statistically significant intergroup differences in flexion contracture, maximum flexion angle, KSKS or KSFS preoperatively or postoperatively (Table 2), though these variables improved significantly in each group postoperatively.

**Table 2:** Comparison of preoperative and postoperative variables between PFC Sigma PS group and RP-F group

Variable	Preoperative		Postoperative	
	PFC Sigma PS group	RP-F group	PFC Sigma PS group	RP-F group
Flexion contracture (°)	8.8 ± 6.39	8.2 ± 6.75	2.0 ± 2.81	1.6 ± 2.78
KSKS	28.5 ± 10.76	26.6 ± 9.87	90.7 ± 5.05	92.2 ± 3.74
KSFS	14.3 ± 8.40	15.4 ± 11.20	76.7 ± 9.71	77.6 ± 7.90
ROM (°)	82.7 ± 16.99	83.0 ± 10.5	110.7 ± 9.67	112.8 ± 4.58
Maximum flexion angle (°)	85.6 ± 17.10	91.2 ± 12.68	114.62 ± 9.82	114.4 ± 5.64

Values presented as mean ± standard deviation (SD)

*RP-F* rotating platform high-flexion, *KSKS* Knee Society knee score, *KSFS* Knee Society function score

° refers to degree in which the angles are measured

Based on Knee Society radiographic criteria, there was no evidence of prosthetic loosening or failure in this cohort. There were four knees in the PFC Sigma PS group and two knees in the RP-F group that had nonprogressive radiolucent lines of less than 2 mm in zone 1, beneath the medial tray, as seen in the AP radiograph obtained immediately postoperatively. However, there were radiolucent lines of less than 2 mm in all zones in one patient from the RP-F group who was suffering from rheumatoid arthritis at 6-month follow-up; on clinical examination, there was no sign of implant loosening. However, there was no further increase in radiolucent line on further follow-up.

Patella resurfacing was done in 20 of 60 cases in the PFC Sigma PS group and 30 of 50 cases in the RP-F group. All the resurfaced patellae in both groups were fixed well without any signs of loosening at final evaluation. No infection occurred and no revision was required in any of the cases. No subluxations or dislocations of any bearing were seen.

## Discussion

There is little doubt that TKA has revolutionized care of patients with end-stage arthritic conditions of the knee by providing significant relief of pain, improving function, and restoring quality of life. However, the expectation of achieving higher flexion after TKA surgery, which can eventually help in carrying out daily activities in Asian population, led to the introduction of high-flexion knee prostheses having improved knee kinematics in high flexion [13]. The PFC Sigma RP-F system, which reduces the radius of curvature of the posterior femoral condyles offset and rotation of the bearing surface, improving internal rotation of the tibia for high flexion of the knee, is one such prosthesis. However, the benefits of these designs with regard to increased postoperative flexion are controversial and still being debated [14].

We undertook this study to analyze whether this high-flexion implant actually improves clinical ROM and function in comparison with fixed-bearing prosthesis, taking into account the excellent results of fixed-bearing prosthesis and cost-effectiveness. Results of the present study suggest that the early clinical outcomes for knees with RP-F prosthesis were similar to those for knees with a standard PCL substituting prosthesis. In both groups, our patients had improved quality of life in terms of pain, walking distance, flexion deformity, and Knee Society clinical and function scores after total knee arthroplasty.

Pain score improved significantly (*p* < 0.0001) in both groups postoperatively, but we found no statistically significant difference between the two groups. Specifically, there was no difference between the groups in terms of the overall KSS or the individual pain, function, and stair-climbing KSS at any point during the follow-up period. These findings are consistent with those in literature, where no significant difference in pain scores has been found between the two implant groups [3, 7, 15, 16].

ROM was measured by clinical goniometer with the patient in supine position. The mean range of flexion preoperatively, at 3 months postoperatively, and at 6 months and yearly thereafter did not differ significantly between the two groups. Our study demonstrated that the improvement of flexion from preoperative values after surgery was significant in both groups. It must be mentioned that our cohort showed no significant difference in preoperative flexion range also, which supports the notion that preoperative flexion range is a major determinant of further postoperative flexion depending on the prosthetic design. Dennis et al. [9] reported that various other factors such as surgical technique, knee kinematics, complications, and postoperative therapies influence postoperative knee flexion apart from implant design. This might possibly explain why implant design alone has rarely shown a difference in postoperative knee flexion [17, 18]. In our study, at 1 year postoperatively, the average gain in flexion was 2.05° more in the RP-F group as compared with the PCL substituting TKR group, although this was statistically insignificant. Literature also suggests that no prospective, randomized control studies have shown a statistical difference in maximum postoperative flexion or ROM. In these studies, the mean flexion ranged from 106° to 130° for the standard design and 110° to 128° for the high-flexion design [14, 19–21]. Our results for the kinematics lie in the range of these studies, and there was no difference in maximum knee flexion between the groups at any follow-up point. However, there are many short-term follow-up studies showing that the flexion angle and maximum flexion angle are 27° greater after TKA using the Sigma RP-F model than TKA using conventional implants [22–25]. Suh et al. [25] reviewed the results of 41 cases of TKA using Sigma RP-F or Sigma PS for a mean period of 26.7 months. The mean maximum flexion angle was greater in the Sigma RP-F group (130.4°) than the Sigma PS group (123.3°), whereas no significant intergroup difference was observed in the clinical outcomes. Similarly, Kim et al. [10] also reported the results on 45 knees that underwent TKA with PFC-Sigma RP-F and PFC Sigma PS, where the RP-F mobile system facilitated greater maximum flexion angle and ROM gain compared with the PS fixed model.

Literature results comparing high-flexion and posterior stabilized designs continue to remain inconclusive and are probably design and surgeon dependent [26]. Two systematic reviews have reported comparisons of conventional posterior stabilized and high-flexion TKA implants. A metaanalysis performed by Gandhi et al. [27] concluded that high-flexion implant designs improve overall range of motion compared with traditional implants but offer no advantage in KSS in primary TKAs. A systematic review by Murphy et al. [28] suggested that there was insufficient evidence to support that high-flexion TKA implants improved range of motion or functional performance. Recently, Sumino et al. [29] conducted a systematic literature review analyzing the change in range of knee flexion from preoperative values following conventional PS and high-flex PS TKA and concluded that improvement of preoperative flexion after TKA using HF prostheses is similar to that of conventional PS prostheses. However, they also suggested that, in the Western patient group, high-flexion implants showed slightly greater improvement of preoperative flexion compared with conventional PS implants. For Asian population, the authors reported no significant difference between the improvement of preoperative knee flexion with standard PS versus high-flex PS implants.

In the current study, we could not find significant differences between the groups regarding clinical scores or maximum range of motion during flexion. The results of our study also suggest that, at almost 6.5 years after of surgery, flexion, range of motion, and KSS were similar in the two groups.

In the earlier part of this study, we did not carry out patella resurfacing regularly, and few of these patients complained of anterior knee pain. After reviewing literature on patella resurfacing, we found that there are few studies which favor patellar resurfacing while a few other studies report worst results following patellar resurfacing. However, some studies reported that patella resurfacing showed similar pain and function scores as compared with non-resurfaced patella in TKA [30]. A few studies also suggest selective patellar resurfacing [31]. In a published decision analysis, the revision rate following primary TKA for patellofemoral problems was 2.8% for resurfaced patella compared with 7.2% for nonsurfaced patella [32]. We also started resurfacing patella and observed that incidence of anterior knee pain decreased in resurfaced patients.

Implant durability or fixation was also assessed on the basis of radiographic appearance. In the present study, although nonprogressive radiolucent lines were observed in four cases in the FB group and two cases in the RP-F group, the overall results were good without any presence of symptoms or complications, including component loosening or polyethylene wear. Cho et al. [33] reported that radiolucent lines were observed in 13.8% of the cases between 3 and 6 years after high-flexion TKA, 3.2% of which required reoperation at mean of 49 months postoperatively. In our study, however, no cases required reoperation or revision.

Currently, methodological limitations and inconsistent results in high-flexion TKA research along with uncertain long-term survivorship [18] as well as the results of the present study suggest no potential benefits in terms of postoperative ROM or function when using these implants. The design also requires resection of an additional 2–4 mm of bone from posterior femoral condyles, which may weaken the bone supporting the load from the femoral component [34]. The downsides of high-flexion designs such as increased cost and increased bone resection limit their use in comparison with conventional PS implants.

In conclusion, based on the results of the present study, we conclude that early clinical and radiographic outcomes, and patient satisfaction rates were similar in the RP-F and PS fixed model groups. Moreover, the gain of 2.05° mean range of motion in the RP-F group was statistically insignificant; it did not translate into any major advantage in terms of clinical outcome or function. Hence, in this population, the increased cost of the RP-F implant was not justified.
